# A Small-Sample Classification Strategy for Extracting Fractional Cover of Native Grass Species and Noxious Weeds in the Alpine Grasslands

**DOI:** 10.3390/s24206571

**Published:** 2024-10-12

**Authors:** Zetian Ai, Ru An

**Affiliations:** 1 School of Geographic Information and Tourism, Chuzhou University, Chuzhou 239000, China; 2 Anhui Province Key Laboratory of Physical Geographic Environment, Chuzhou University, Chuzhou 239000, China; 3 School of Geography and Remote Sensing, Hohai University, Nanjing 211100, China; anrunj@163.com

**Keywords:** alpine grasslands, fractional cover extraction, feature optimization method, training sample extension algorithm, composite three-kernel SVM classifier

## Abstract

The fractional cover of native grass species (NGS) and noxious weeds (NW) provides a more comprehensive understanding of grassland health in the alpine grasslands. However, coverage extraction of NGS and NW from satellite hyperspectral imagery can be challenging due to the small spectral and spatial feature difference, insufficient training samples, and the lack of effective fractional cover extraction methods. In this research, firstly, a feature optimization method is proposed to optimize the difference feature between NGS and NW. Secondly, a spectral–spatial constrained re-clustering training sample extension method (SSCTSE) is proposed to increase the number of training samples. Thirdly, a composite three-kernel SVM method (CTK-SVM) is developed to produce fractional cover maps of NGS and NW. The experimental results show that (1) the feature optimization method is effective in preserving the spectral and spatial difference features while eliminating invalid features; (2) the SSCTSE algorithm is capable of significantly increasing the number of training samples; (3) the fractional cover maps of NGS and NW are produced with the CTK-SVM method with overall accuracies of approximately 65%, and the RMSEs of NGS and NW are approximately 16% and 11%, respectively. The results provide a foundation for the fractional cover extraction of different grass species in alpine grasslands based on satellite hyperspectral imagery.

## 1. Introduction

According to the research published in Nature Reviews Earth and Environment, grassland degradation is widespread in alpine grassland due to human and natural factors, such as extensive overgrazing and an increase in rodent populations, seriously affecting the sustainable use of alpine grasslands [1]. In China, the alpine grasslands are a geographically extensive ecosystem that is distributed across the Three-River Headwater Region (TRHR), which is situated in the hinterland of the Tibetan Plateau [2]. Due to the harsh natural conditions (such as high altitude, a cold climate, and scarce oxygen), alpine grasslands are characterized by short growth and low cover, and have been prone to grassland degradation. Grassland degradation is mainly manifested in two aspects [3]: Firstly, there is a reduction of total grassland coverage and biomass. Secondly, there is a change of grassland structure; that is, the coverage of native grass species (NGS) decreases and that of noxious weeds (NW) increases. At present, remote sensing techniques to extract the status of alpine grasslands mainly focus on total grassland coverage [4] and biomass [5,6]. These studies are unable to obtain the changes of grassland structure and the coverage of NGS (which is important for livestock farming) and cannot effectively monitor the productivity and health of the alpine grasslands [3]. Therefore, it is necessary to monitor the changes of grassland structure, i.e., to monitor the coverage of NGS and NW, to obtain information on the productivity and health status of alpine grassland.

The NGS in the alpine grasslands mainly include *Kobresia* and *Stipa purpurea*, and the NW mainly include *Saussurea nivea*, *Gowardesh flowers*, *Ajania tenuifolia*, *Leontopodium nanum* and *Whin*, etc. Currently, the study of identification of NGS and NW mainly focuses on three aspects: (1) The identification of grass species in the alpine grasslands based on canopy spectral measurement data [7,8], which demonstrated that the spectra of NWS and NW are slightly different; (2) The identification of NGS and NW based on the UAV hyperspectral imagery [9], which show that NGS and NW can be recognized in hyperspectral and high-spatial resolution images; (3) identification of NGW and NW based on satellite hyperspectral images [10,11], which show that recognizing NGS and NW in satellite hyperspectral images have a certain feasibility. In conclusion, the recognition of NGS and NW in the alpine grasslands has a certain research basis. However, there is a relatively small number of studies on the fractional cover extraction of NGS and NW, especially using UAV or satellite hyperspectral images.

The alpine grasslands in the TRHR are a vast area with high altitudes and low oxygen levels, making field sampling challenging. Due to the sparse, short, and mixed growth of grassland, the spectral differences between different grass species are not significant on low spatial and low spectral resolution imagery, making it challenging to effectively identify NGS and NW from satellite hyperspectral imagery. In fact, the fractional cover extraction of NGS and NW is constrained by three key challenges. Firstly, the spectral and spatial difference features of NGS and NW based on satellite hyperspectral images must be identified. Secondly, the lack of field samples must be addressed. Thirdly, effective extraction methods for alpine grasslands must be developed. At present, the fractional cover extraction of NGS and NW based on satellite hyperspectral images has two methods: Multiple Endmember Spectral Mixture Analysis (MESMA) [10] and classification [11]. In fact, the classification of hyperspectral images with small samples has proven to be a challenging task [12], representing one of the difficulties in the practical application of hyperspectral imaging [13].

Satellite hyperspectral images are capable of distinguishing the spectral and spatial differences between different types of vegetation. They have been widely used for the fine classification of vegetation type [14] and natural grassland cover. Hyperspectral images derived from satellite or airborne platforms can identify sugar cane species [15], invasive reed vegetation [16], and sericea lespedeza [17]. Furthermore, six different vegetation types can be identified from seagrass communities [18]. With regard to the fine classification of natural grasslands, the grasslands of the Northern Tibetan Plateau can be classified into five different types [19]. However, due to the complexity of vegetation growth, the recognition accuracy is higher for vegetation with regular growth, while it is slightly lower for vegetation with mixed growth.

The classification of hyperspectral images with small samples needs to solve the problems of insufficient samples, high dimensionality of hyperspectral data, and small differences between different features. Three processing steps are usually used to solve the above problems. First, the training samples are expanded. To augment the number of samples, supervised classification, model prediction, and a posteriori probability are utilized to facilitate the label passing [20,21]. Second, the feature dimensions are reduced to extract the optimal features. For example, the hyperspectral image is decomposed into low-rank signals to remove noise, and then the main features are extracted using the PCA transform [12]. Third, the appropriate classification and regression methods are employed to obtain the final categories [22]. In order to enhance the separability of the feature, the kernel transform is employed to map the features to a high-dimensional space [23,24].

This research builds on the previous paper [11] and go-further works as follows: (1) proposed a new feature optimization method for class separability of grasslands with different cover levels, and solved the feature optimization problem of NGS and NW; (2) proposed a spectral–spatial constrained re-clustering training sample extension method (SSCTSE), and solved the problem of insufficient training samples of alpine grasslands; (3) in terms of the spectral and spatial difference features of NGS and NW, a composite three-kernel SVM (CTK-SVM) method was developed. Based on the above methods, the fractional cover maps of NGS and NW were produced.

## 2. Materials and Methods

### 2.1. Study Area and Hyperspectral Image

#### 2.1.1. Study Area

**Figure 1:**
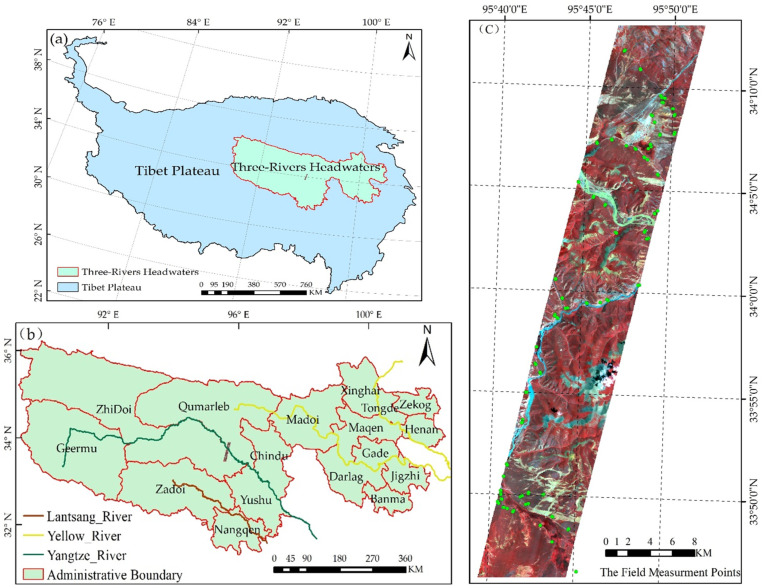
Study area. (**a**) The location of the TRHR on the Qinghai–Tibetan Plateau. (**b**) The location of the study area in the TRHR. (**c**) Hyperion image of the study area and the field measurement points.

The study area is situated in the middle of the Three-River Headwater Region (TRHR), which is the largest alpine grasslands nature reserve in China. The TRHR is the headwaters of the Yangtze, Yellow, and Lantsang Rivers in the hinterland of the Qinghai-Tibetan Plateau (see Figure 1a). The TRHR is characterized by alpine grasslands, which account for approximately 76.2% of the total area. The study area is located in the middle of the TRHR and is in the central region of Qumarleb County, as shown in Figure 1b. The altitude of this area range is approximately 4200 m above sea level, and the average annual temperature is between −6 and 4 °C. The main grass species are *Kobresia humilis*, *Potentilla chinensis*, *Astragalus* sp., *Leontopodium nanum*, *Ajania*, etc. The study area is typical of the TRHR in terms of its location, altitude, and grass species, and is an ideal grazing area for yaks. Figure 1c shows the Hyperion hyperspectral image of the study area.

#### 2.1.2. Hyperspectral Image

The Hyperion data selected for this study is of the L1GST class, which has been radiometrically corrected, geometrically corrected, projectionally aligned, and topographically corrected. The image was taken on 3 August 2012, and covered parts of Zhiduo and Qumalai counties. The pre-processed Hyperion hyperspectral images of the study area are shown in Figure 1c. Although the Hyperion hyperspectral sensor is no longer operational, its spectral and spatial characteristics remain representative, and the associated findings can be utilized in extant hyperspectral imagery.

The growth cycle of grass species in the study area is from May to September. According to the temperature and precipitation data from 2013 to 2019 in the research area from the National Meteorological Information Center of China: http://data.cma.cn/site/index.html (accessed on 11 August 2024), the temperature and precipitation change rates during the growth period in the study area were both around 5%. Studies show that the fluctuation rate of fractional vegetation coverage (FVC) based on MODIS data in the TRHR is about 4% [11], and the overall trend becomes better [25]. In addition, research has shown that the inter-annual changes of grasslands in the TRHR were relatively small [26]. The reason is that there is slow growth of grassland and small changes in grassland habitats on a plateau that is over 4000 m above sea level. Therefore, although there was a gap of several years between the sampling data and Hyperion image, the sampling data can still reflect the actual grassland coverage status.

### 2.2. Field Investigation

#### 2.2.1. Sample Data Collection

The time of the field investigation was in early August 2017 and 2019, which was the flourishing period for the alpine grasslands. During the field investigation, the canopy spectra of typical grass species were measured, and the grassland coverage samples were collected.

**Figure 2:**
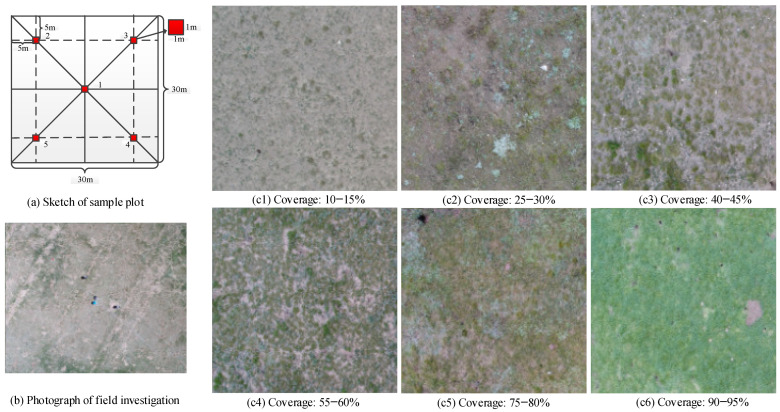
Sketch maps of grassland samples and various coverage levels: (**a**) Sketch of sample plot; (**b**) Photograph of field investigation; (**c**(**1**–**6**)) Photographs are the grassland coverage levels.

The dimensions of each sample site were defined as 30 m × 30 m, which was the same as the resolution of the Hyperion image. At each sample site, five small quadrats, each with a size of 1 m × 1 m, were established and distributed in an “X” shape, as shown in Figure 2a. The use of the “X” shape sampling scheme mainly aimed to improve the sampling efficiency because most representative sample sites are relatively homogeneous (see Figure 2c). During the field investigation, a Global Positioning System (GPS) receiver with a positional accuracy of less than 1 m was first used to obtain the latitude and longitude at the center of the sample site on the frame of a WGS84 coordinate system (quadrat 1 in Figure 2a). Then, the quadrats were photographed from above in order to document their surrounding environment. Furthermore, the grass species present in each quadrat were identified and their coverage was estimated by visual inspection of three persons. Moreover, all grass species were classified into two types: NGS and NW. Finally, the overall coverage of NGS and NW of each sample site was obtained by averaging the NGS coverage and the NW coverage of the five quadrats. A total of 118 field samples were collected within the study area (Figure 1c).

#### 2.2.2. Vegetation Spectral Survey

**Figure 3:**
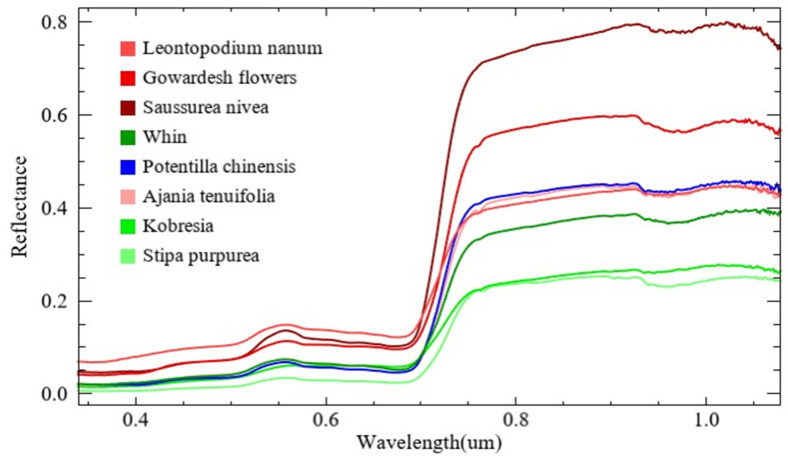
The original spectral curves of typical vegetation were measured in the study area.

Some canopy spectra of representative grass species were measured using the spectrum-measuring instrument HR-1024, manufactured by the SVC Company in the USA. The measurement time range was from 11:00 to 14:00 on sunny days. For one grass species, five sampling points with coverages of nearly 100% were selected for spectral measurement. A total of 10 spectra for one grass species were collected, and then the mean spectral reflectance value was calculated in the ViewSpecPro 5.6 software as the final spectrum. Eight typical grass species spectra including *Kobresia*, *Stipa purpurea*, *Saussurea nivea*, *Gowardesh flowers*, *Ajania tenuifolia*, *Leontopodium nanum*, *Potentilla chinensis*, and *Whin were collected*. The spectral curves of the grass species were showed in Figure 3.

In this study, the NGS mainly include *Kobresia* and *Stipa purpurea*, which account for more than 95% of the NGS coverage. The remaining grass species belong to the NW, with more than 90% of NW comprising *Leontopodium nanum*, *Ajania tenuifolia*, and *Whin*.

The reference [27] shows that the spectral curves of different grassland coverages show significant differences in the hyperspectral images. In this research, the grass cover of NGS and NW was grouped into ten levels as follows: 0 ≤ C < 10, 10 ≤ C < 20, 20 ≤ C < 30, 30 ≤ C < 40, 40 ≤ C < 50, 50 ≤ C < 60, 60 ≤ C < 70, 70 ≤ C < 80, 80 ≤ C < 90, and 90 ≤ C < 100 (C is the percentage of grass coverage). The NGS and NW with different coverage levels should be distinguished based on their spectral difference. The number of field samples of NGS and NW are shown in Table 1. The number of field samples totaled 118, of which 60 were training samples, and 58 were validation samples.

### 2.3. Feature Extraction and Optimization Method

#### 2.3.1. Difference Feature Extraction Method

(1). Spectral difference features

Based on the canopy spectra of NGS and NW, their first-order derivative spectra and continuum removal spectra were calculated [28]. The spectral difference bands between NGS and NW were extracted using the Mahalanobis distance method [29] based on their canopy spectra, first-order derivative spectra, and continuum removal spectra. The Mahalanobis distance method is an effective method for identifying the spectral differences between different vegetation types and has been widely used in identification of tree and grass species [30]. When selecting spectral difference bands using the Mahalanobis distance method, two principles should be followed: Firstly, the Mahalanobis distance value of the difference bands should be higher than the average value. Secondly, the selected spectral difference bands should be larger than the minimum spectral resolution of the hyperspectral image used (Hyperion images should be larger than 10 nm).

(2). Vegetation index features

Vegetation indices are widely used for qualitative and quantitative estimations of vegetation parameters [31], including vegetation cover, biomass, and chlorophyll content [32]. It has been shown that inversion values based on vegetation indices are able to discriminate between different grassland cover levels [32] and even invert the composition of biochemical components in different vegetations [33]. Therefore, the following three types of vegetation indices were selected as features for this study: (1) vegetation indices for estimating vegetation cover, including NDVI, nGNDVI, and nNDVI; (2) vegetation indices for estimating chemical components, including PRI, nPRI, nLCI, VOG2, and ARI2; and (3) vegetation index regulating atmospheric influence, VARI.

(3). Spatial texture difference features

Due to the natural characteristics of short growth and mixing of different grass species in the alpine grasslands (the spatial texture features of the grassland are not obvious in remote sensing images), hyperspectral images of the alpine grasslands show a random and irregular texture structure. Therefore, this research tries to extract both texture and morphological features of the grassland in order to test their spatial difference features on NGS and NW. Texture features are extracted using a Gabor filter, which can extract texture features at different scales and directions in the frequency domain [34]. This filter has been widely used for spatial feature extraction from hyperspectral images [35,36]. Morphological features are extracted using the Extended Morphological Profile (EMP), which is capable of extracting morphological features of an image using structural elements of different sizes [37]. This morphological feature has been shown to improve the classification accuracy of hyperspectral images [38,39,40].

#### 2.3.2. Feature Optimization Method

The aim of this research is to identify the cover levels of NGS and NW; the grassland with different cover levels is an independent class, and therefore the separability of grassland with different cover levels needs to be investigated. The commonly used method to discriminate the class separability is the J–M distance method, but this method has two obvious drawbacks in discriminating the separability of grasslands with different cover levels. Firstly, it needs to calculate the distance of grasses with different cover levels on each difference feature, which is a larger computational effort when there are more difference features and more computational data. Secondly, the interval values of grasses with different cover levels on each difference feature are discretized, and further integration, comparison, and visualization are required, which is a vast amount of work.

This research proposes a novel class separability feature optimization method to solve separability problems of grasslands with different cover levels in feature space. This method has the advantages of small computation, clear quantification, and strong visualization. The method is described as follows:

(1). The spectral values corresponding to the field sampling points are extracted from the difference features; these values form a data matrix.
(2). The data matrix is classified according to different cover levels, the average spectral values of each cover level are obtained, and the average spectral curves of different cover levels are obtained.
(3). The spectral curves of different cover levels are plotted and the differentiation of the spectral curves of different cover levels is checked.
(4). The bands with the smallest intervals in the spectral curves should be deleted in order to obtain the final features.

**Figure 4:**
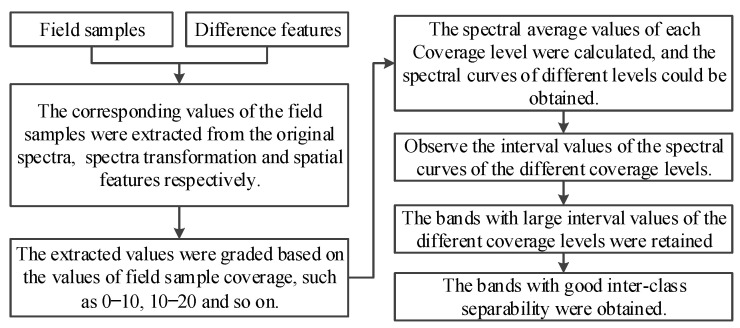
A feature optimization method for class separability of grasslands with different cover levels.

The technical flow of the method is shown in Figure 4.

### 2.4. Training Sample Extension Method

Two principal methods exist for the expanding of training samples. Firstly, expanding new training samples near the existing field samples. For example, training samples can be extended in a hyper-pixel, where all pixels of the hyper-pixel are designated as training samples [13]. Secondly, new training samples can be extended from the results of the supervised and semi-supervised classification results. For example, the training samples can be extended based on the posterior probability [21,41]. Given the natural characteristics of the NGS and NW in the alpine grasslands, neither of the above methods is applicable. The first method is to select the training samples that are close to each other, suitable for the region of pure pixels. If the training samples are all surrounded by pure pixels, the extended samples are likely to be of the same class. This method can be used if grassland is considered to be in the same class, but this method is not suitable for this research. The second method is to obtain classification results first and select training samples later. However, if the training samples are insufficient, the accuracy of grassland fine classification is poor and the extended training samples are considered inappropriate, so this method is not suitable for application in this paper. Therefore, it is necessary to develop new sample extension methods that are suitable for grasslands.

This research is based on two basic common-sense principles: First, spatially neighboring pixels can have the same class label. Second, pixels with similar spectral signals can have the same class label. Consequently, this research extends new training samples from the surrounding areas of the field samples [26] and presents a spectral–spatially constrained re-clustering training sample extension (SSCTSE) method. Firstly, Euclidean Distance is used as an initial constraint to select alternative samples within a specified range surrounding the field samples. Secondly, Spectral Angle Distance [42] (SAD) is used as the second constraint to calculate the spectral difference between the alternative samples and the field samples. The alternative samples with minimal spectral differences are retained. Thirdly, the retained alternative samples are analyzed by the Fuzzy C-means algorithm (FCM), and then the alternative samples that are clustered together and close to the field sample are retained. Finally, the selected alternative samples are added to the training samples. The specific flow of the method is shown in Table 2.

The advantages of this method are as follows: the first step ensures the spatial proximity of the extended samples, the second step ensures the spectral similarity of the extended samples, and the third step ensures that the spectral vectors between the extended and the field samples are positively correlated, avoiding the situation where the spectral angles are the same but the spectra are different.

### 2.5. Composite Three-Kernel SVM Method

The SVM method is able to achieve high classification in the condition of high-dimensional data and limited samples. In addition, it is able to process data at a faster rate, making it an optimal choice for remote sensing images with large areas. The integration of the SVM method with the spectral–spatial composite kernel has been demonstrated to improve classification accuracy. This approach was first proposed by Li [43] in 2013. Fang [44] developed the mothed it in 2015, and then Ranjan [45] modified it in 2019. The above studies have shown that a composite three-kernel SVM method based on multiple features can significantly improve the classification accuracy over a two-kernel SVM [46]. There are three types of difference features of NGS and NW, namely original spectra, mathematically transformed spectra and spatial features. Therefore, this research proposes the construction of a composite three-kernel SVM (CTK-SVM) method, which includes the original spectral kernel, the mathematically transformed spectral kernel, and the spatial feature kernel. The aim is to achieve optimal classification results by adjusting the parameter weights of each kernel function.

(1). SVM mothed

Let $(X_{1},X_{2},\cdots X_{N})\in R^{M}$ is training samples, its corresponding category label is $\left(C_{1},C_{2},\cdots C_{N}\right)$, SVM mothed mainly solves the following problems.

(1) $$\underset{w,\beta_{i},b}{\min}\left\{\frac{1}{2}{\left\|w\right\|}^{2}+T\sum_{i}\beta_{i}\right\}$$

Subject to $c_{i}\left(\langle Ø(x_{i},w)\rangle +b\right)\geq 1-\beta_{i},{and\beta }_{i}\geq 0$.

Where, w,b is a feature space classifier, $\beta_{i}$ is the relaxation variable, T is a regularization parameter, $Ø(x_{i},w)$ represents a mapping function and can map the pixel $X_{i}$ to the high dimensional space $Ø(x_{i})$. The mapping function of the SVM is an inner product function, usually represented by the Radial Basis Function (RBF).

(2) $$K\left(X_{i},X_{j}\right)=exp(-{\left\|X_{i}-X_{j}\right\|}^{2}/2\sigma^{2})$$

where, σ is the parameter of the kernel function.

Substituting formula (2) into (1), we obtain the decision criteria for each pixel by solving a Lagrange problem:

(3) $$f\left(y\right)=\sum_{i=1}^{N}c_{i}\alpha_{i}K\left(X_{i},X_{j}\right)+b$$

where, $\alpha_{i}$ is the Lagrange factor, which can be solved by the quadratic optimization method.

(2). Three-kernel construction method

Let $X_{i}$ is a pixel, its spectral characteristic is $X_{i}^{g}$, its corresponding mathematically transformed spectral characteristic is $X_{i}^{b}$, its corresponding spatial information is $X_{i}^{s}$, we can construct a composite three-kernel function:

(4) $$K\left(X_{i},X_{j}\right)=\left[K^{g}\left(X_{i}^{g},X_{j}^{g}\right),K^{b}\left(X_{i}^{b},X_{j}^{b}\right),K^{s}\left(X_{i}^{s},X_{j}^{s}\right)\right]$$

where, $K^{g}\left(X_{i}^{g},X_{j}^{g}\right)$ represents the spectral feature kernel, $K^{b}\left(X_{i}^{b},X_{j}^{b}\right)$ represents the transform spectral feature kernel, $K^{s}\left(X_{i}^{s},X_{j}^{s}\right)$ represents the spatial feature kernel.

Furthermore, weights are assigned to the three-kernel functions to construct a spectral, transformed spectral, and spatial composite kernel.

(5) $$K_{gbs}\left(X_{i},X_{j}\right)=\mu_{g}K^{g}\left(X_{i}^{g},X_{j}^{g}\right)+\mu_{b}K^{b}\left(X_{i}^{b},X_{j}^{b}\right)+\mu_{s}K^{s}\left(X_{i}^{s},X_{j}^{s}\right)$$

where, $\mu_{g}$, $\mu_{b}$ and $\mu_{s}$ represent the weights of the spectral, transformed spectral and spatial feature kernels, respectively, and $\mu_{g}+\mu_{b}+\mu_{s}=1$. The composite kernel formula (5) can be substituted directly into (3).

(3). Three-kernel construction method of training samples

In actual calculations, the corresponding training samples also need to construct spectral, transformed spectral and spatial feature kernels: $K^{g,train}\left(X_{i}^{g,train},X_{j}^{g,train}\right)$,$K^{b,train}\left(X_{i}^{b,train},X_{j}^{b,train}\right)$ and $K^{s,train}\left(X_{i}^{s,train},X_{j}^{s,train}\right)$. Set the weights for the three-kernel functions to construct a composite three-kernel of training samples.

(6) $$K_{gbs}^{train}\left(X_{i},X_{j}\right)=\mu_{g}K^{g,train}\left(X_{i}^{g,train},X_{j}^{g,train}\right)+\mu_{b}K^{b,train}\left(X_{i}^{b,train},X_{j}^{b,train}\right)+\mu_{s}K^{s,train}\left(X_{i}^{s,train},X_{j}^{s,train}\right)$$

where, $\mu_{g}$, $\mu_{b}$ and $\mu_{s}$ represent the weights of original spectral, transformed spectral and spatial feature kernels of the training samples, respectively, and $\mu_{g}+\mu_{b}+\mu_{s}=1$. In actual operation, the weights of spectral kernel, transformed spectral kernel and the spatial kernel of the training samples are consistent with those in formula (5).

### 2.6. The Technical Flow Chart of the Small-Sample Classification Strategy

**Figure 5:**
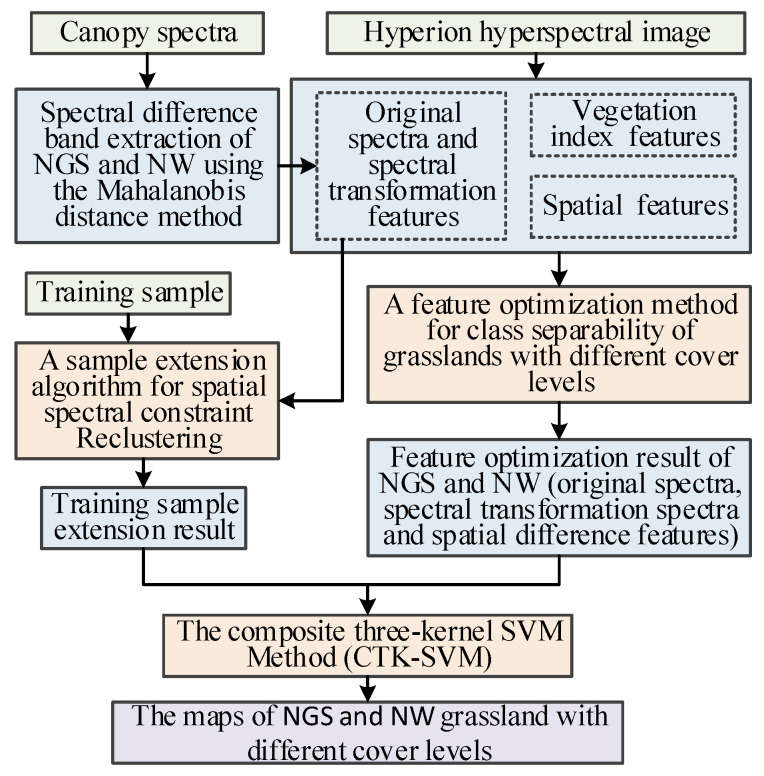
Technical flow chart.

The small-sample classification strategy for extracting fractional cover of the NGS and NW mainly include five stages: (1) Image pre-processing, (2) Features extraction and optimization, (3) Training samples extension, (4) The composite three-kernels SVM method, (5) Fractional cover maps of NGS and NW. The flowchart is shown in Figure 5.

## 3. Results and Discussion

### 3.1. The Results of Feature Extraction and Optimization

#### 3.1.1. The Difference Features of NGS and NW

(1). Spectral difference feature

The canopy spectral difference ranges of NGS and NW were obtained based on the Mahalanobis distance method, as shown in Table 3. Then, the corresponding original, continuum removal and first-order derivative bands were extracted from the Hyperion hyperspectral image based on the canopy spectral difference range, as shown in Table 3.

(2). Vegetation indices feature

The characteristics of the vegetation indices used are shown in Table 4.

(3). Spatial difference feature

Principal Component Analysis (PCA) was applied to the Hyperion image difference features (35 bands) to extract the first two PCA components, and then the Gabor features and EMAP features were calculated, respectively, based on these two bands. The parameters were set as follows: the wavelength scale of the Gabor function was set to two, the standard deviation of both the direction angle and the Gaussian factor was set to 0.5, and the direction was set to 30, 60, 90, 120, 150, and 180. Each PCA component generated 6 Gabor features, for a total of 12 features. The EMAP radius was set to 1, the step size was set to 2, the number of openings and closings was set to 4, and each PCA component formed 9 EMAP features, for a total of 18 features.

#### 3.1.2. The Optimization Results of Difference Features

**Figure 6:**
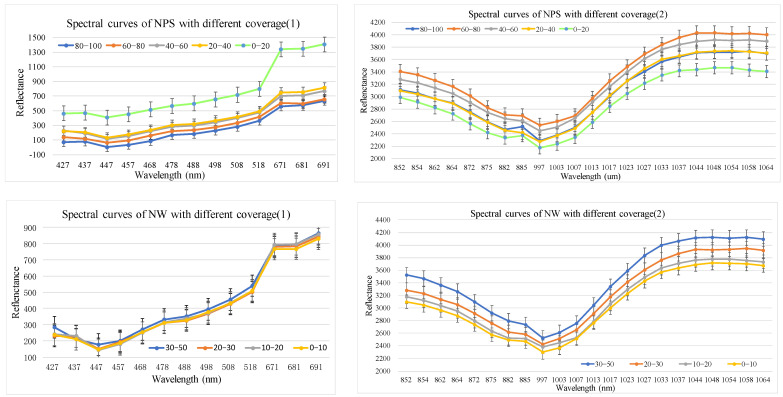
Spectral curves of NGS and NW with different coverage levels.

**Figure 7:**
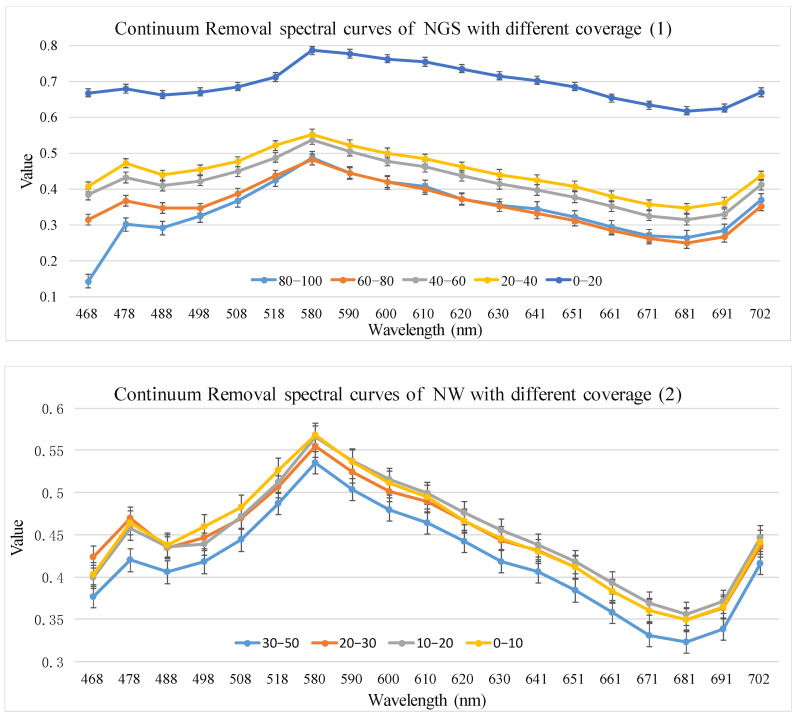
Continuum Removal spectral curves of NGS and NW with different coverage levels.

**Figure 8:**
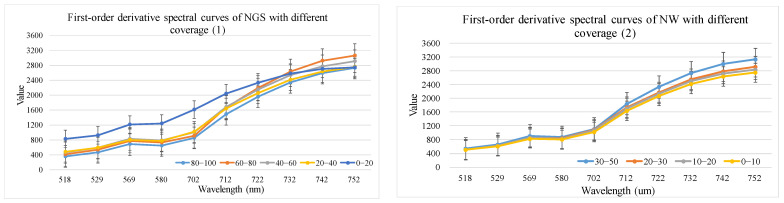
First derivative spectral curves of NGS and NW with different coverage.

**Figure 9:**
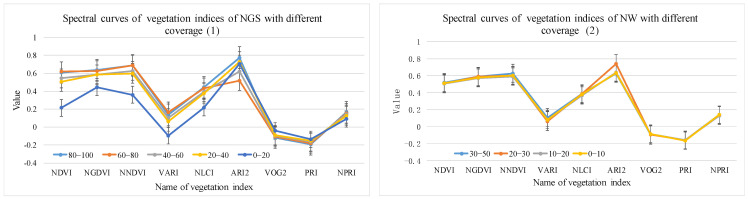
Vegetation index spectral curves of NGS and NW with different coverage.

**Figure 10:**
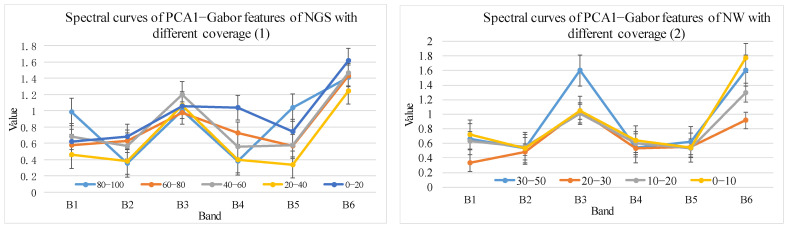
PCA–Gabor features of NGS and NW with different cover levels.

**Figure 11:**
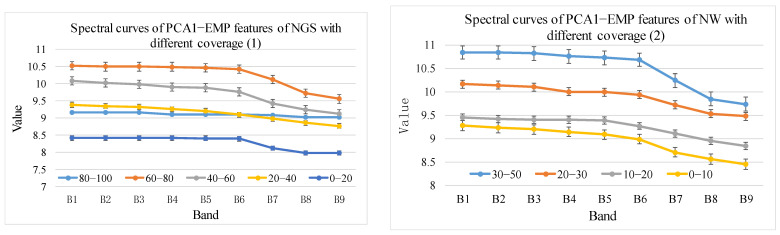
PCA1–EMP features of NGS and NW with different cover levels.

Using the method in Section 2.3.2, spectral profiles with different cover levels were obtained based on 35 Hyperion difference bands, 19 envelope bands, 10 first-order derivative bands, 9 vegetation indices, 12 Gabor features, and 18 EMAP features. The corresponding results are shown in Figure 6, Figure 7, Figure 8, Figure 9, Figure 10 and Figure 11.

(1). Spectral feature optimization results

As shown in Figure 6, the NGS with different cover levels exhibited some differentiation in the visible region within 477.7–691.4 nm, but the two levels of 20–40% and 40–60% were indistinguishable. In the near infrared region, the four levels (0–20%, 20–40%, 40–60%, 60–80%) were clearly distinguishable in the difference band. However, the level of 80–100% showed some inconsistencies due to the limited number of field samples and the occasional larger errors in this class. The NW with different cover levels were indistinguishable in the visible region, but clearly distinguishable in the NIR.

(2). Continuum Removal feature optimization results

As shown in Figure 7, the envelope spectra of the NGS showed obvious spectral difference in the wavelength range of 467.5–508.2 nm and 671–701.6 nm. The envelope spectra of the NW showed no spectral difference in the preferred bands.

(3). First-order derivative feature optimization results

As shown in Figure 8, the first-order derivative spectra of the NGS showed some spectral differences in the wavelength range of 518.4–701.5 nm. The first-order derivative spectra of NW showed some differences in the wavelength range of 711.7–752.4 nm.

(4). Vegetation index feature optimization results

As shown in Figure 9, the vegetation index values of NGS differed slightly for NDVI, NGDVI, NNDVI, and VAVI, and did not differ for the other five vegetation indices. The vegetation index values of NW did not differ for the nine vegetation indices.

(5). Texture features optimization results

Based on Figure 10, there is no difference in the Gabor values of NGS and NW in different cover levels, which cannot be used to discriminate the cover information of NGS and NW.

As shown in Figure 11, the EMP values of the first five bands exhibit notable differences for the NGS, which can be used to differentiate NGS with different cover levels. However, errors occur in the 80–100% level. NW were clearly distinguished in all nine EMP bands. The EMP values based on the second PCA component were not clearly differentiated and could not be used to distinguish NGS and NW, thus they are not shown here.

In summary, the difference features of NGS and NW with different cover levels are shown in Table 5.

### 3.2. The Results of Training Sample Extension

The training samples were extended in an area of 5 × 5 pixels centered on the field samples. The spectral threshold was set to less than 0.045 for NGS and less than 0.030 for NW. The extended samples were clustered using the fuzzy C-means algorithm, with the clustered points centered on the field samples retained as the extended training samples. The sample extension results are shown in Table 6 and Table 7.

### 3.3. The Fractional Cover Maps of GNS and NW

#### 3.3.1. Accuracy Verification

Based on the difference features of NGS and NW (Table 5), the fractional cover of NGS and NW were extracted using the extended training samples (Table 6 and Table 7) and the composite three-kernel SVM method, which constructs the original spectral kernel, spectral transform kernel, and spatial feature kernel. A total of 58 field samples were used to validate the extraction accuracies of NGS and NW. Two methods were used for statistical accuracy: first, the number of coverage differences between the estimated and measured values that were less than 10, 15, and 20; and second, the root mean square error (RMSE) between estimated and measured values. For ease of calculation, the middle value of the coverage level is taken as the standard value. For instance, if the cover class is 0 ≤ C < 10, the middle value is 5.

#### 3.3.2. Map of NGS with Different Coverage Levels

**Figure 12:**
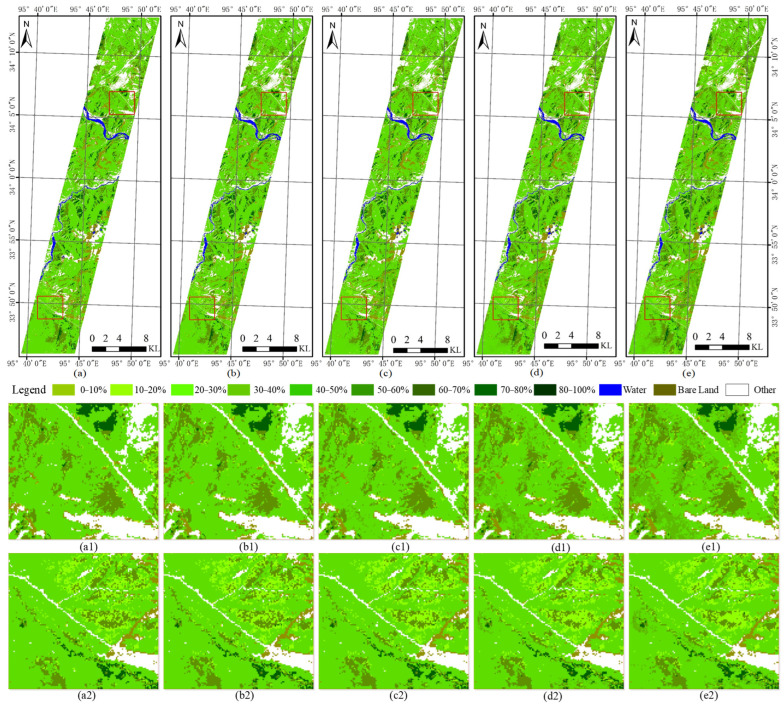
The fractional cover maps of NGS with different weight ratios. The weights of (**a**–**e**) are 0.4:0.4:0.2, 0.5:0.4:0.1, 0.6:0.3:0.1, 0.7:0.2:0.1, and 0.8:0.1:0.1, respectively. The enlarged areas of the upper red box in (**a**–**e**) are **a1**, **b1**, **c1**, **d1** and **e1**. The enlarged areas of the lower red box in (**a**–**e**) are **a2**, **b2**, **c2**, **d2** and **e2**.

Because of the band ratios of original spectral, transformed spectral, and spatial features is 30:18:5, the weight ratios of the composite three-kernels were tested: 0.4:0.4:0.2, 0.5:0.4:0.1, 0.6:0.3:0.1, 0.7:0.2:0.1, and 0.8:0.1:0.1, respectively. The corresponding results are shown in Figure 12a–e. As shown in Figure 12, the resulting maps of the five different weighting ratios are generally similar. As the weighting of the spectral features increases, the detailed features of the resulting maps gradually become more pronounced and refined, with the most detailed information observed in the D and E maps. Consequently, it can be seen that the original spectral features are of greater importance in the fractional cover extraction of NGS and NW.

The results of the validation accuracy of NGS based on 58 field samples are presented in Table 8. The verification accuracies of the 0.4:0.4:0.2 combination and the 0.5:0.4:0.1 combination are higher than those of other combinations. The percentage of estimated and measured values differed by less than 10, 15, 20, and 25, and accounted for 46.5%, 58.6%, 65.5%, and 79.3% of the total validation samples, respectively. Moreover, the RMSE between the estimated and measured values is approximately 17%. It can be concluded that the optimal extraction accuracy is achieved with the three-kernel weight ratios of 0.4:0.4:0.2 and 0.5:0.4:0.1, which corresponds to the band ratio of 30:18:5 for the three feature types.

#### 3.3.3. Map of NW with Different Coverage Levels

**Figure 13:**
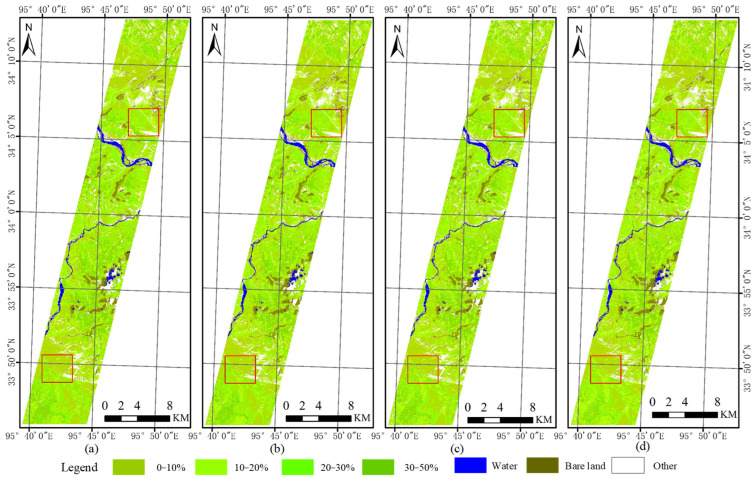

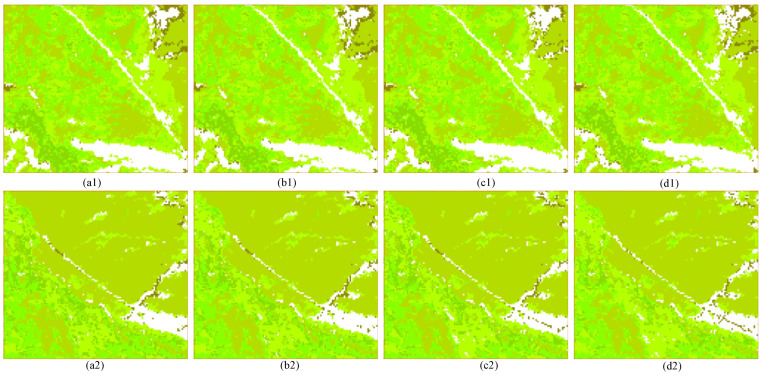
The fractional cover maps of NW with different weight ratios. The weights of (**a**–**d**) are 0.5:0.2:0.3, 0.6:0.2:0.2, 0.7:0.1:0.2, and 0.8:0.1:0.1, respectively. The enlarged areas of the upper red box in (**a**–**d**) are **a1**, **b1**, **c1**, and **d1**. The enlarged areas of the lower red box in (**a**–**d**) are **a2**, **b2**, **c2**, and **d2**.

Because the band ratios of original spectral, transformed spectral and spatial features is 22:5:9, the weight ratios of the composite three-kernel were tested: 0.5:0.2:0.3, 0.6:0.2:0.2, 0.7:0.1:0.2, and 0.8:0.1:0.1, respectively. The corresponding results are shown in Figure 13a–d. As shown in Figure 13, the four maps exhibit a high degree of similarity, primarily due to the relatively low cover of poisonous weeds. In contrast to these maps, the 0.7:0.2:0.1 and 0.8:0.1:0.1 combinations, which are weighted more heavily with original spectral information, are relatively more detailed and accurate.

The results of the validation accuracy of the NW based on 58 field samples are presented in Table 9. The verification accuracy of the 0.5:0.2:0.3 combination is higher than that of other combinations. The percentage of estimated and measured values differed by less than 5, 10, and 15, accounted for 43.1%, 58.6%, and 79.3% of the total validation samples, respectively. Moreover, the RMSE between the estimated and measured values is approximately 10.9%. It can be concluded that the optimal extraction accuracy is achieved with the three-kernel weight ratios of 0.5:0.2:0.3.

### 3.4. Discussion

#### 3.4.1. The Impact of Feature Reduction on Recognition Accuracy

Considering the large number of optimized features, which may contain redundant features and affect the computational efficiency in practical applications, the research tested the extraction accuracy of NGS by feature reduction using half and third features, respectively. The first retains one band for every two features, for a total of 28 bands. The second retains one band for every three features, for a total of 19 bands. In the experiment, the weight ratio of spectral kernel, spectral transform kernel, and spatial kernel was set to 0.5:0.4:0.1. The training and validation samples were kept consistent in the upper subsection.

The validation accuracies based on 58 field samples are shown in Table 10. The validation accuracies of the feature reduction are close to those of the full bands, but the details of the fractional maps are gradually lost. The experiments also demonstrate the effectiveness of the difference features and the stability of the composite three-kernel SVM method.

The experiments show that appropriate feature reduction does not reduce the overall accuracy. However, the feature reduction based on the mathematical transformation method, such as principal component analysis (PCA), is not applicable to fractional cover extraction. The extraction maps and the accuracy validation are all inferior. The primary reason for this is that the grass spectral features are situated in a low-dimensional space. The feature reduction based on mathematical transformation has the effect of destroying the fine characteristics of hyperspectral images and reducing the differentiation of grassland with different coverage levels.

#### 3.4.2. The Main Factors Affecting the Recognition Accuracy

The fractional cover extraction accuracy of NGS and NW can be affected by three factors.

(1). Field samples

The high altitude and hypoxic geographical characteristics of the alpine grasslands, coupled with the natural characteristics of mixed grass species and low cover, have resulted in the quantity and quality of field samples with different cover levels being insufficient, especially with the lack of field samples with pure pixels, which is one of the main factors affecting the extraction accuracy of NGS and NW. Although the number of training samples is increased by the sample extension method proposed in this research, the quality of these extension samples is limited, which also affects the extraction accuracy of NGS and NW.

(2). Different features

The experiments demonstrated that original spectral and spectral transformation difference features played a key role in the coverage extraction of NGS and NW. However, due to the low cover of alpine grasslands, the spectral information of satellite images of both NGS and NW is weak, which affects the accuracy of the cover extraction. In fact, the spectral differences between NGS and NW with different coverage levels are minimal, and it is challenging to identify their distinguishing features. Moreover, there is a lack of suitable optimization methods for the selected difference features, thus this research proposes a novel feature optimization method that is suitable for fractional cover of grassland.

(3). Identification methods

The fractional cover extraction of NGS and NW is more challenging than that of overall grass coverage. The most commonly used methods, including the pixel dichotomy method, maximum likelihood method, and spectral angle classification, are principally applicable to the extraction of overall grass coverage [47,48]. However, these methods are not suitable for the fractional cover extraction of NGS and NW. Due to the limited number of field samples of alpine grasslands, deep learning methods are also not applicable for this task. The SVM and sparse representation methods are more effective in handling high-dimensional small-sample data and achieving higher accuracy, making them suitable for the fractional cover extraction of NGS and NW. However, the sparse representation method is less efficient and practical when dealing with high-dimensional data, especially when using the kernel function [49]. Therefore, the SVM method is a relatively good method for the coverage extraction of alpine grasses. In this research, the SVM method was developed based on the original spectra difference, transformed features difference, and spatial features difference of NGS and NW, and a multi-feature composite three-kernel SVM method was constructed. The objective was to advance the application of the SVM method in alpine grasslands classification.

## 4. Conclusions

In this research, the fractional cover maps of NGS and NW in the alpine grasslands were produced based on satellite hyperspectral imagery. This research reached the following conclusions.

(1). A new feature optimization method for class separability of grasslands with different cover levels was proposed. Based on this method, the difference features of NGS and NW were optimized, and the difference feature ranges of original spectra, spectral transformations, and spatial features of NGS and NW were further reduced. The method is also applicable to the optimization of difference features for other grass species.
(2). A new spectral–spatial constrained re-clustering training sample extension method was proposed. This method is able to effectively increase the number of training samples by adjusting the spatial distance and spectral angle. Furthermore, it is able to exclude the samples with large errors by re-clustering. This method is also applicable to the extension of training samples when classifying similar vegetation types.
(3). A composite three-kernel SVM method was constructed, which includes an original spectral kernel, a spectral transformation kernel, and a spatial feature kernel. Based on the mothed, the fractional cover maps of NGS and NW were produced, and the overall accuracies are approximately 65%. The RMSE of NGS and NW is approximately 16% and 11%, respectively.

The findings of this study not only support the fractional cover extraction of NGS and NW at the regional scale of the alpine grasslands, but also provide a foundation for fractional cover extraction of grass species types in alpine grasslands based on satellite imagery.

## Figures and Tables

**Table 1:** The number of field samples of the NGS and NW.

Grassland Coverage Level	Number of the Training Samples		Grassland Coverage Level	Number of the Training Samples	
	NGS	NW		NGS	NW
0 ≤ C < 10	15	39	50 ≤ C < 60	15	0
10 ≤ C < 20	33	41	60 ≤ C < 70	33	0
20 ≤ C < 30	46	24	70 ≤ C < 80	46	0
30 ≤ C < 40	19	11	80 ≤ C < 90	19	0
40 ≤ C < 50	5	3	90 ≤ C < 100	5	0

**Table 2:** The SSCTSE method.

**Inputs:** the locations and classes of the field samples, hyperspectral images
The method consists of three steps: **A:** Spatial distance constraints to select extended samples. Based on the location of the field samples, the locations of the 5 × 5 pixels around the field sample are extracted to form the extended pixel set, which is used as the extended samples. **B:** Spectral similarity constraints to select alternative samples. Based on the hyperspectral image, the spectral information of the extended samples is extracted. Then, the spectral angular distances between the field samples and each extended sample are calculated and a threshold (usually less than 0.05) is set to further select the alternative samples. **C:** Intra-class re-clustering to select extended samples. For the same class of alternative samples selected in the second step, the Fuzzy C-mean algorithm is used for re-clustering by setting two cluster centers, the samples of the cluster center containing the field samples are retained, and these samples are used as the final extended samples.
**Output:** The locations, classes, and numbers of the extended samples.

**Table 3:** Spectral difference features of NGS and NW.

Spectral Type	Range of Canopy Spectral Difference	Corresponding Hyperion Imaging Bands	Number of Bands
Canopy spectra	345–523 nm, 853–946 nm, 985–1069 nm.	1–10 (345–523 nm), 44–50 (853–946 nm), 51–64 (985–1069 nm). Blue Valley (671 nm, 681 nm), Green Peak (691 nm)	35
First-order derivative	693–752 nm.	5–10 (460–523 nm), 16–28 (572–705 nm).	19
Continuum Removal	460–523 nm, 572–705 nm.	28–33 (693–752 nm). trilateral parametric band: 518 and 528 nm, 569 and 579.	10

**Table 4:** Vegetation indices feature.

Name	Formula	Name	Formula
NDVI	(R852 − R651)/(R852 + R651)	nLCI	(R850 − R710)/(R850 + R680)
nGNDVI	(R780 − R550)/(R780 + R550)	VOG2	(R734 − R747)/(R715 + R726)
nNDVI	(R800 − R670)/(R800 + R670)	ARI2	R800 × [(R550)-1-(R700)-1]
PRI	(R531 − R570)/(R531 + R570)	VARI	(R555 − R680)/(R555 + R680 − R480)
nPRI	(R550 − R530)/(R550 + R530)		

**Table 5:** Optimization results of NGS and NW features with different cover levels.

Feature	Difference Features of NGS	Difference Features of NW
35 Hyperion hyperspectral bands	477–523 nm (5 bands), 852–885 nm (8 bands), 985–1069 nm (14 bands), Blue Valley (671 nm, 681.2 nm), Green Peak (691 nm). A total of 30 bands.	852–885 nm (8 bands), 985–1069 nm (14 bands). A total of 22 bands.
19 Continuum Removal features	467–508 nm (5 bands), 671–702 nm (4 bands). A total of 9 bands	all unavailable
10 first-order derivative features	518 nm, 529 nm, 569 nm, 580 nm, 702 nm. A total of 5 bands.	712 nm, 722 nm, 732 nm, 742 nm, 752 nm. A total of 5 bands
9 vegetation index features	NDVI, NGDVI, NNDVI, VAVI. A total of 4 bands.	all unavailable
12 Gabor features	all unavailable	all unavailable
18 EMP features	The B1–B5 of EMP features are based on PCA1. A total of 5 bands.	The B1–B9 of EMP features are based on PCA1. A total of 9 bands.

**Table 6:** Sample extension results of NGS based on the SCCTSE method.

Grassland Coverage Level	Number of Training Samples		Grassland Coverage Level	Number of Training Samples	
	Field	Extended		Field	Extended
0 ≤ C < 10	3	16	50 ≤ C < 60	8	80
10 ≤ C < 20	4	22	60 ≤ C < 70	7	62
20 ≤ C < 30	6	50	70 ≤ C < 80	2	24
30 ≤ C < 40	10	76	80 ≤ C < 100	2	31
40 ≤ C < 50	18	118			

**Table 7:** Sample extension results of NW based on the SCCTSE method.

Grassland Coverage Level	Number of Training Samples		Grassland Coverage Level	Number of Training Samples	
	Field	Extended		Field	Extended
0 ≤ C < 10	20	147	20 ≤ C < 30	13	131
10 ≤ C < 20	22	161	30 ≤ C < 50	5	43

**Table 8:** The validation accuracies of NGS with different weight ratios.

Accuracy Index (Estimated and Measured)	Weight Ratios of the Composite Three-Kernel				
	0.4:0.4:0.2	0.5:0.4:0.1	0.6:0.3:0.1	0.7:0.2:0.1	0.8:0.1:0.1
Less than 10	27	27	26	25	24
Less than 15	34	34	33	32	31
Less than 20	38	38	37	37	37
Less than 25	46	46	45	45	45
RMSE	16.94%	17.17%	17.78%	17.89%	17.72%

**Table 9:** Validation accuracies of NW with different weight ratios.

Accuracy Index (Estimated and Measured)	Weight Ratios of the Composite Three-Kernel			
	0.5:0.2:0.3	0.6:0.2:0.2	0.7:0.1:0.2	0.8:0.1:0.1
Less than 10	25	24	24	24
Less than 15	34	33	33	33
Less than 20	46	46	46	46
RMSE	10.87%	11.00%	11.00%	11.00%

**Table 10:** Validation accuracies of NGS with dimensionality reduction.

Accuracy Index (Estimated and Measured)	Half Features	Third Features	Full Features
Less than 10	26	26	27
Less than 15	37	36	34
Less than 20	38	38	38
RMSE	16.70%	16.59%	17.1%

## Data Availability

The Hyperion data can be loaded from the website: http://earthexplorer.usgs.gov (accessed on 11 August 2024). The field sampling data is the data of the project team and is not willing to be shared.
